# Hydrodynamic alignment and assembly of nanofibrils resulting in strong cellulose filaments

**DOI:** 10.1038/ncomms5018

**Published:** 2014-06-02

**Authors:** Karl M. O. Håkansson, Andreas B. Fall, Fredrik Lundell, Shun Yu, Christina Krywka, Stephan V. Roth, Gonzalo Santoro, Mathias Kvick, Lisa Prahl Wittberg, Lars Wågberg, L. Daniel Söderberg

**Affiliations:** 1 Wallenberg Wood Science Center, KTH Royal Institute of Technology, Stockholm SE–100 44, Sweden; 2 Linné FLOW Centre, KTH Mechanics, KTH Royal Institute of Technology, Stockholm SE–100 44, Sweden; 3 Department of Fibre and Polymer Technology, KTH Royal Institute of Technology, Stockholm SE–100 44, Sweden; 4 Photon Science, DESY, Notkestrasse 85, Hamburg D–22607, Germany; 5 Ruprecht Haensel Laboratory, University of Kiel, Kiel D–24098, Germany; 6 Helmholtz-Zentrum Geesthacht, Institute for Materials Research, Geesthacht D–21502, Germany; 7 Innventia AB, PO Box 5604, Stockholm SE–114 86, Sweden

## Abstract

Cellulose nanofibrils can be obtained from trees and have considerable potential as a building block for biobased materials. In order to achieve good properties of these materials, the nanostructure must be controlled. Here we present a process combining hydrodynamic alignment with a dispersion–gel transition that produces homogeneous and smooth filaments from a low-concentration dispersion of cellulose nanofibrils in water. The preferential fibril orientation along the filament direction can be controlled by the process parameters. The specific ultimate strength is considerably higher than previously reported filaments made of cellulose nanofibrils. The strength is even in line with the strongest cellulose pulp fibres extracted from wood with the same degree of fibril alignment. Successful nanoscale alignment before gelation demands a proper separation of the timescales involved. Somewhat surprisingly, the device must not be too small if this is to be achieved.

## Abstract (web summary)

Nanofibrils isolated from wood have potential as building blocks for materials, but in order to obtain high performance, control of the assembly process is needed. Here, the authors report a method to process nanofibrils using microfluidics, giving filaments with aligned fibrils and high strength.

## Graphical abstract

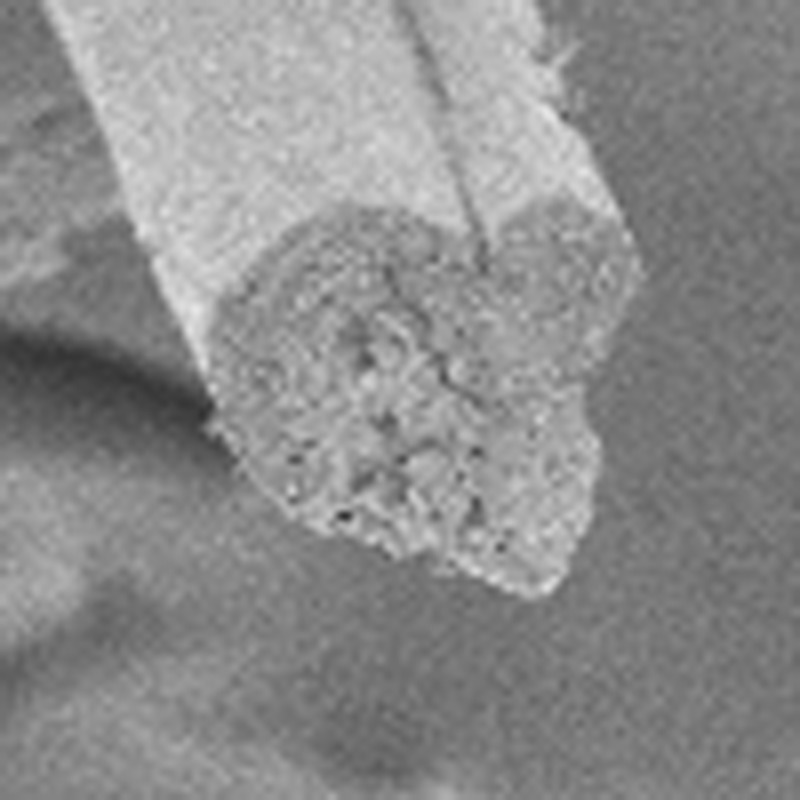

Many biological materials show impressive and controllable properties that are determined by their micro- and nanostructures. Cellulose fibres extracted from wood and silk represent two excellent examples^1^,^2^,^3^. The main constituent of cellulose fibres is the nanoscale fibril, which has the prospective of being a building block for future high-performance biomaterials and textiles^4^,^5^ and/or provide a template for functional nanomaterials^6^. However, processes that enable full utilization of the potential of the fibrils are yet to be developed. Fibrils in cellulose fibres from wood are organized in a nanoscale lamellar structure having a highly ordered spiralling orientation along the fibre axis^7^. The fibres demonstrate high ultimate strength and stiffness that vary in a wide range depending on the mean fibril orientation^2^,^4^,^5^,^8^,^9^,^10^. In the tree, the fibril orientation also varies through the thickness of the stem so that the mechanical performance of the tree is optimized^10^. Cellulose fibres can be disintegrated^11^,^12^ into individual fibrils or fibril bundles (cellulose nanofibrils, CNF) and, recently, films and filaments have been manufactured from CNF^5^,^6^,^13^,^14^,^15^,^16^. However, the properties obtained are far from the values reported for individual cellulose fibres liberated from wood^2^,^10^ and it can be hypothesized that the fibrils have to be aligned and assembled in a controlled manner in order to make use of the potential of CNF.

We have successfully designed a continuous and potentially industrially scalable and parallelizable method that prepares strong and stiff CNF-based filaments. We have also identified critical mechanisms and associated timescales that govern our filament-forming processes, as well as the necessary separations of these timescales needed for successfully replicating the properties of the natural cellulose fibre. The process is realized using a millimetre-sized flow-focusing system^17^,^18^,^19^,^20^ as the primary component and the identified mechanisms and associated timescales are generic and will govern similar assembly processes of shape-persistent anisotropic substances—for example, other types of fibrils, fibroins or even organic polymers during injection moulding^21^. For the case of non-shape-persistent particles the timescale for shape relaxation in the channel must be added and tuned to ensure that assembly occurs before the particle relaxes. Nevertheless, the reasoning may readily be applied to processes for microfluidic assembly of, for example, silk^18^,^22^. As long as the timescales of the alignment and assembly process are correct, up or downscaling and parallelization of this process for industrial production are possible. This will allow manufacturing of strong filaments from wood fibre raw material for future production of high-performance bio-composites as well as for textile production. In the latter context, the filaments could be a replacement product for cotton and industrially produced viscose and Lyocell, and thereby significantly contribute to a reduced environmental footprint by reduced use of organic solvents.

## Results

### Mechanical performance of the CNF filaments

**Figure 1:**
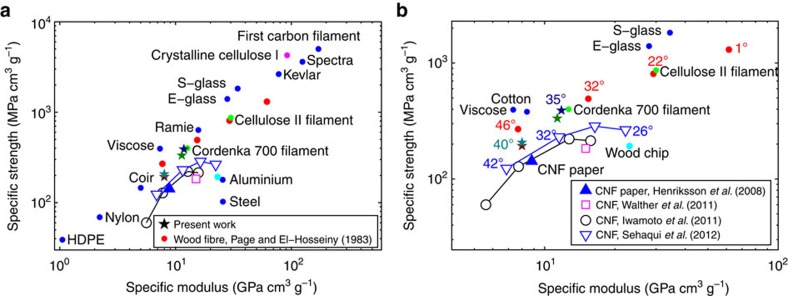
Properties of common filament materials. (**a**,**b**) Overview and close-up of specific ultimate strength versus specific Young’s modulus for a number of materials, respectively. Solid red dots represent measurements of cellulose pulp fibres extracted from wood, while the open markers are filaments and films made of CNF. The properties of filaments prepared by alignment followed by dispersion–gel transition are represented by filled stars, where the four different colours belong to four different cases. The angle (in °) displayed next to some markers in **b** represent the CNF alignment (if this information was given), where zero is in the direction of the fibre or filament axis. Further details are given in the text.

The obtained CNF filaments have been evaluated regarding fibril orientation, stiffness, ultimate strength and strain-to-failure. In Fig. 1 (overview in 1a and close-up in 1b), our filaments (filled stars) are compared with the specific ultimate strength as a function of specific Young’s modulus for a wide range of filament materials as well as steel and aluminium^4^,^7^,^23^,^24^. The filled, red markers show data that have been obtained from stress–strain curves for bleached cellulose pulp fibres extracted from wood^2^ assuming a fibre density of 1.3 g cm^−3^. More recent experiments report lower values^10^ and the red circles should therefore be considered to be extremely good values. The red circles correspond to different angles between the mean fibril orientation within the fibre and the fibre orientation (nanofibril angle); this variation occurs naturally since the tree optimizes its structural integrity. The data points for cellulose pulp fibres follow the trend given by most of the other fibres ranging from plastic fibres in the lower left, via natural fibres to stronger and stiffer synthetic fibres such as glass-, Kevlar-, Spectra- and carbon fibres in the upper right. Note that cellulose pulp fibres with fully aligned fibrils can have a specific ultimate strength comparable to glass fibres and a specific stiffness comparable to Kevlar. The two green filled circles show (one of) the strongest commercially available filaments made from dissolved cellulose (Cordenka 700) as well as the strongest cellulose filament ever reported^24^.

The open connected markers (square, triangles and circles) and the filled triangle show properties of filaments and films made from CNF^14^,^15^,^16^,^25^. These filaments have been made by the ejection of a CNF dispersion from a nozzle (syringe) followed by coagulation^14^,^15^ and the fibril alignment is a function of the ejection speed. The fibril orientation in these previously reported results is averaged over the filament width (~100 μm) and it is therefore not possible to deduce whether the fibril orientation varies from the skin to the core. However, scanning electron microscopy (SEM) images of these filaments show that the cross-section varies considerably and that the filaments often are hollow. It should be noted that the CNF used to prepare our filaments were produced with the same protocol as the CNF used to prepare the film represented by the filled triangle. However, our filaments reproduce the properties of the cellulose pulp fibres as well as the strongest commercially available filaments made from dissolved cellulose. Previous man-made CNF-based materials are thus far from this achievement.

### The concept for filament assembly

**Figure 2:**
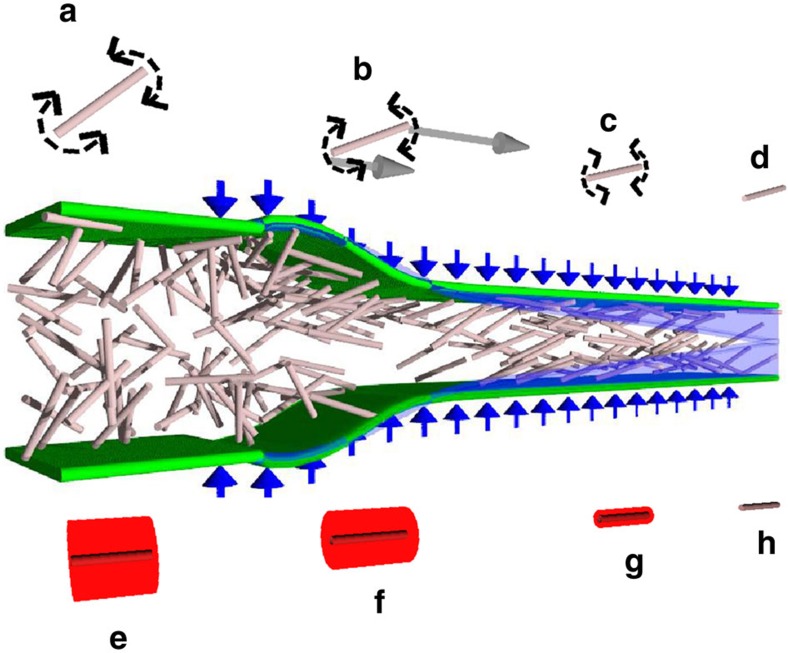
Illustration of the assembly process. The nanofibrils in the focused flow are illustrated as rods (the fibril length in relation to the channel width is exaggerated by approximately a factor of 300). The diffusion of Na ^+^ , from addition of NaCl in the focusing liquid, is illustrated with a blue tint. The rows of small images above and below the central image illustrates the hydrodynamical, molecular and electrochemical processes involved. (**a**) Brownian diffusion (illustrated with the dashed arrows) affects the orientation of a single fibril, (**b**) hydrodynamically induced alignment (illustrated by solid, grey, arrows) occurs during the acceleration/stretching, (**c**) Brownian diffusion continues to act after the stretching has ceased, (**d**) Brownian diffusion is prevented by the transition to a gel. The lower row of small images illustrate how the electrostatic repulsion (illustrated by the red area representing the Debye length) decreases from **e** to **h** as the Debye length is decreased with increasing Na^+^ concentration or by protonation of the carboxyl groups on the fibril surface.

Our filaments are prepared by utilizing a surface-charge-controlled gel transition^26^,^27^ in combination with hydrodynamically induced fibril alignment. Ideally, the assembly phase of the filament-forming process should first align the fibrils in the dispersion before fixing the material nanostructure by inducing a gel transition. Figure 2 shows an idealized description of our concept of achieving this. Above and below the illustration of flowing nanofibrils, the mechanical (above) and electrochemical (below) processes affecting the fibrils are illustrated. In the liquid dispersion, fibrils are fairly free to rotate (a), thanks to strong electrostatic repulsion (e). An accelerating flow causes the fibrils to align in the flow direction^20^,^21^,^28^ (b). Before the alignment is lost due to Brownian diffusion (c), the electrostatic repulsion between the particles is reduced by an electrolyte diffusing into the suspension (f–h). Finally, the aligned structure is frozen as a gel (d).

Hydrodynamical alignment can be achieved in different ways. The cross-section of the flow channel can be increased or decreased, imposing deceleration or acceleration, respectively, of the flow. As a consequence, fibrils will tend to orient themselves perpendicular (deceleration) or parallel (acceleration) to the flow direction^20^,^21^,^28^. This could be referred to as geometry-controlled acceleration and it occurs in spinnerets as well as in spinning using a syringe^14^,^15^,^29^. For the latter case, the velocity gradients towards the walls will inevitable impose a continuous rotational motion of the fibrils, even though the mean orientation will be aligned with the flow direction. This rotational motion in shear has been shown also for nematic liquid crystals^30^.

For the case of wet-spinning, the fibril dispersion would be injected into an outer co-flowing liquid—that is, the sheath flow. The sheath flow commonly has a higher speed than the core stream; however, it can also have a lower speed. This velocity difference will induce shear that accelerates or decelerates the stream with fibrils and hence affects fibril alignment^31^. However, for this case the shear is omnipresent and causes changes of the mean fibril orientation as well as the orientation distribution. Another possibility is to use two perpendicular opposing (focusing) streams that merge with the core (focused) fibril dispersion stream. For this case, the acceleration is controlled by continuity, which gives a minimum of shear. This flow set-up is often referred to as flow focusing.

### Filament assembly using flow focusing

**Figure 3:**
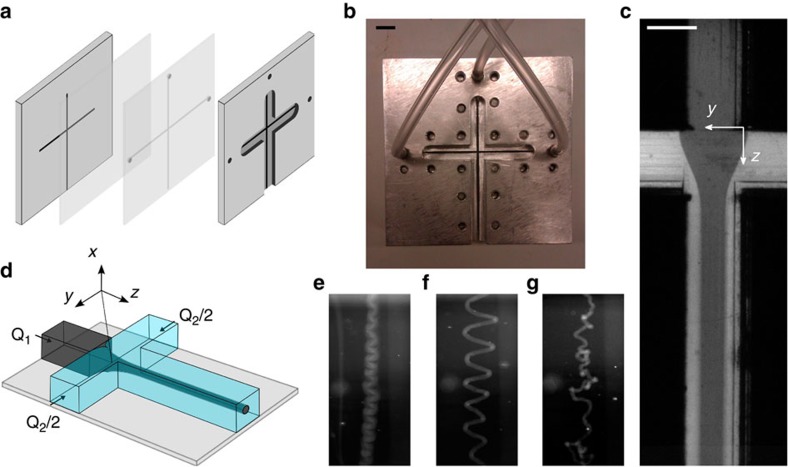
Description of experimental set-up. (**a**,**b**) Schematic drawing and photo of the flow cell, respectively, where the scale bar in **b** is 10 mm. (**c**) Image of the focusing region of the channel, where the flow is directed downwards and the scale bar represents 1 mm. Water is focusing an ink–water mix. (**d**) A schematic drawing of the flow focusing part. (**e**–**g**) Images of the ejected jet, where water is focusing CNF in **e** and a NaCl solution is used to focus CNF in **f**,**g**. A higher acceleration is used in **g** compared with **f**.

In our experiments, alignment followed by gelation is accomplished in a flow-focusing channel system^17^,^18^,^19^,^22^ seen in Fig. 3. The flow is illustrated in Fig. 3c. If the outer (or sheath) streams contain electrolytes or an acid, ions will diffuse into the dispersion causing a gel transition^26^,^27^ at positions where the ion concentration has reached values above the gelation concentration threshold. This gel transition is because of a cancellation of the electrostatic repulsion between the fibrils, which originates from the carboxyl groups on the fibrils. The result is the formation of a gel thread at the centreline of the channel, and in order to avoid buckling the flow in the channel must be hydrodynamically stable. This is an example of a viscous confined jet/wake flow and the buckling is due to a hydrodynamic instability causing self-sustained oscillations^32^,^33^, which can be avoided by selecting the proper flow parameters.

After forming, the gel thread is ejected for further processing. The parameters of this ejection (viscosity of the liquid and velocities) must be in a range where the gel thread exits from the conduit in an orderly manner, without entanglement of the thread and/or destruction of the gel. Figure 3f shows successful ejection. The cases in Fig. 3e,g result in threads that cannot be dried; in Fig. 3e the sheath liquid is deionized water by which no phase transition occurs and in Fig. 3g the thread buckles because of an elevated sheath flow rate.

In the present work, filaments formed at four different process conditions are presented, see Table 1. Case B is our baseline case. Case A is ejected into deionized water instead of NaCl solution. Case C is formed with a lower sheath flow concentration of NaCl (later gelation) and case D is formed with less acceleration (less alignment during acceleration).

**Figure 4:**
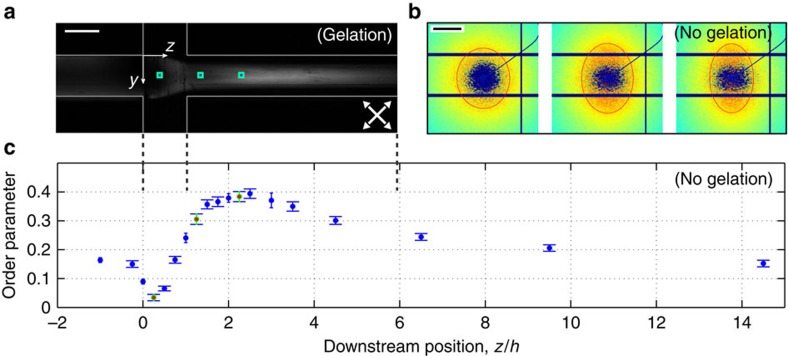
Alignment and de-alignment of fibrils in the channel. (**a**) A NaCl solution is focusing an CNF dispersion. The channel is placed between two crossed polarization filters rotated 45° from the vertical axis (white arrows). (**b**) SAXS diffractograms from before, during and after the acceleration by pure water in the channel, where the red ellipse corresponds to a constant intensity. The locations are marked with green squares around blue markers in **a**. (**c**) Order parameter from acceleration by pure water calculated from SAXS data as a function of downstream distance normalized with the channel width *h*=1 mm. The error bars represent the s.d.’s between different *q*-values. The scale bar in **a** is 1 mm and in **b** 0.5 nm^−1^.

The alignment process in the flow-focusing device is visualized by polarized light in Fig. 4a. In our case the birefringence of the CNF dispersion results in higher intensity of the transmitted light in regions where the fibrils are aligned. The alignment is further demonstrated using small-angle X-ray scattering (SAXS) in Fig. 4b (qualitative) and 4c (quantitative). The SAXS images in Fig. 4b are taken from the positions marked with green squares around blue markers in Fig. 4a and the red contours show that the initially isotropic structure (circle) is deformed further downstream. This deformation in small-angle diffraction is a footprint of alignment on the nano level.

From the SAXS images, the local order parameter can be calculated (see Methods). The variation in the order parameter along the channel length for one flow rate ratio (cases A, B and C) is shown in Fig. 4c, where the horizontal scale is the same as in Fig. 4a. In these measurements, deionized water is used as sheath liquid, which means that there is no gelation, and order parameter 0 represents an isotropic fibril orientation distribution, whereas order parameter 1 represents a fully aligned fibril orientation distribution—that is, all fibrils are aligned in the filament direction. Initially, the shear in the inlet channel (*z*/*h* <0) creates order in the incoming dispersion. At the start of the focusing (*z*/*h*≈0), the flow geometry is such that there is a deceleration causing fibril de-alignment (order decreases). This decrease in order is followed by a steep increase in order because of the acceleration (the maximum order reached is around 0.39). After this increase, Brownian motion causes a slow relaxation towards isotropy further downstream (*z*/*h*>2.5).

In order to create a homogeneous and smooth gel thread from a dispersion of elongated particles or molecules (fibres, fibrils, polymers or carbon nanotubes) in the channel with a high order of alignment, the structure must be assembled into a gel after alignment has been achieved but before Brownian diffusion causes relaxation towards isotropy. Furthermore, complete gelation must have been reached before the material is convected/transported out of the channel system. As a consequence of these conditions, the relations between four timescales representing the fibril alignment, ion diffusion into the thread, Brownian rearrangement of the fibrils and the convection through the device must be correct.

### Timescales controlling the assembly process

The timescales involved can be readily identified and estimated. The alignment of the fibrils is achieved through the acceleration caused by the flow focusing. Given the square cross-section of the channel with area *h*^2^, the volumetric flow rate of the focused fluid, *Q*_1_, and an estimate for the length of the acceleration observed in the images, ~2*h*, the timescale of this process is *t*_align_~2*h*^3^/*Q*_1_. Here *Q*_1_=6.5 mm^3 ^s^−1^ and *h*=1 mm, which gives *t*_align_~0.31 s.

In order to create a gel network and lock the aligned structure, the ion concentration must increase inside the CNF thread through diffusion and the related timescale is given by *t*_ion_~*Ch*^2^/*D*_ion_, where *D*_ion_ is the diffusivity. In this expression, *C* is a constant given by the flow conditions, geometry, outer concentration and transition concentration. The value can be estimated by solving the diffusion equation in cylindrical coordinates


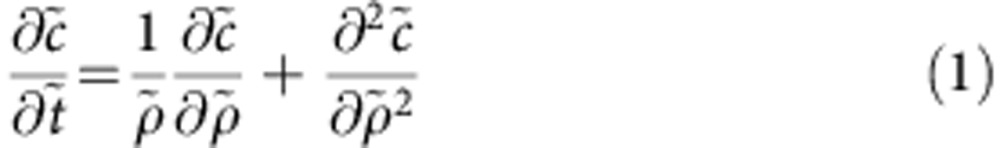


where the thread centre coincides with the longitudinal axis of the cylindrical coordinate system and diffusion is considered only in the radial direction. The diffusion equation has been non-dimensionalized by *h*, *D*_ion_ and initial outer concentration and can be solved numerically. This was carried out in MATLAB on the domain 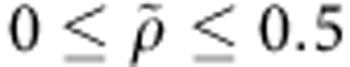 with 
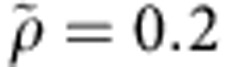
 as an estimate of the thread radius. No-flux and symmetry boundary conditions were imposed at 
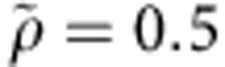
 and 0, respectively. The initial conditions were 
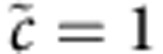
 outside and 
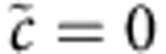
 inside the thread. In order for the gelling to start, the concentration must be over ~10 mM. Thus, given an initial outer concentration of 100 mM the thread should be completely gelled when 
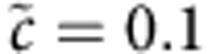
 at the centreline, which gave 
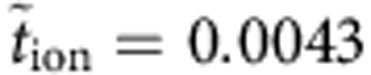
. With *D*_ion_=1.3 × 10^−9^ m^2 ^s^−1^ for Na^+^ in water, this gives the dimensional timescale 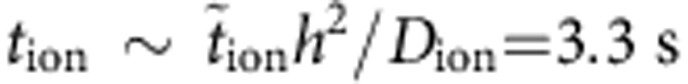.

Before the structure in the thread is locked through gelling, there will be a Brownian rearrangement of the fibrils towards isotropy, which is a diffusive process with a rotational diffusivity *D*_rot_ (ref. 34). The timescale of this process is given by *t*_rot_~Δ*φ*^2^/*D*_rot_ where Δ*φ* is the maximum de-alignment that can be accepted. However, the diffusivity constant *D*_rot_ is difficult to estimate from first principles and has therefore been measured as described in the Methods section. By choosing Δ*φ*=22.5° as the maximum de-alignment and by using the measured *D*_rot_=0.04 rad^2 ^s^−1^ this gives *t*_rot_~3.9 s.

Finally, the thread needs to be kept intact during gelation while convected out through the channel. This convective timescale is given by *t*_conv_~*Lh*^2^/(*Q*_1_+*Q*_2_), where *L* is the length of the outlet channel and *Q*_1_, *Q*_2_ are the volumetric flow rates of the core and sheath flows, respectively. For our case, *Q*_1_=6.5 and *Q*_2_=7.5 mm^3 ^s^−1^, and *h*=1 mm and *L*=50 mm, which gives *t*_conv_~3.6 s.

It should be noted that the timescales are independent of the viscosities, which would not be the case if the acceleration was induced by shear from a sheath liquid at higher speed. Furthermore, an important aspect of the timescales is that they scale differently with respect to channel size and flow rate. Thus, these two parameters can be altered in order to achieve the necessary separation of the timescales.

In order to achieve a successful assembly of our fibrils into an anisotropic filament, the conditions on these timescales are *t*_align_<*t*_ion_<*t*_rot_ and *t*_ion_<*t*_conv_. We achieve this in cases A and B since our flow conditions give the estimates *t*_align_=0.31 s, *t*_ion_=3.3 s, *t*_rot_=3.9 s and *t*_conv_=3.6 s. In the case C, *t*_ion_ is increased (approximately doubled) because of the decreased concentration of ions in the sheath flow and, consequently, the fibrils should de-align more before gelling. Finally, the sheath flow rate (and thus the acceleration) is decreased in case D. As a consequence, the maximum alignment in the channel should decrease compared with cases A–C. For these cases, the development of the fibril order without gelation is shown in Fig. 4c. The estimates show that the margins in terms of timescale separation are distinct but less than one order of magnitude.

### Structural and mechanical characterizations of the filaments

**Figure 5:**
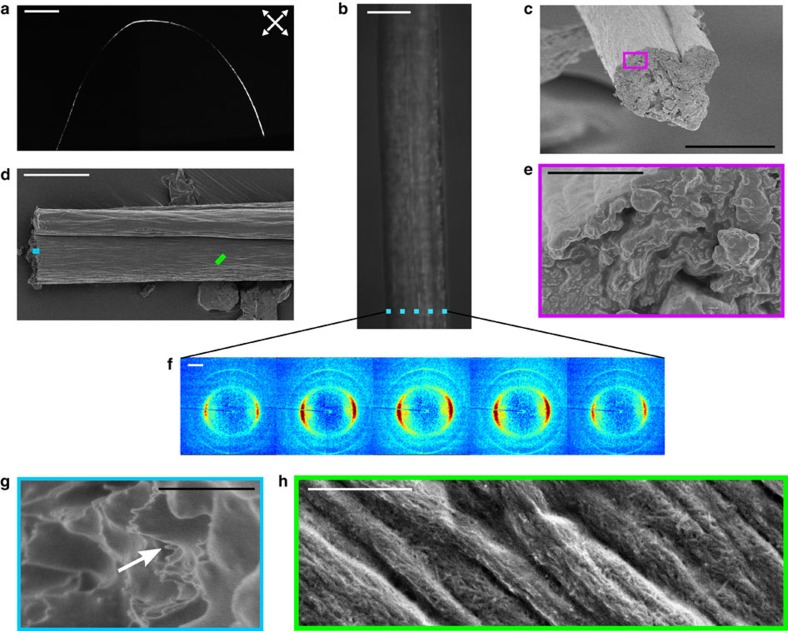
Images and diffractograms of dried filament from case B. (**a**) Image of a single filament placed between two crossed polarization filters rotated 45° with respect to the vertical axis (white arrows), the scale bar represents 10 mm. (**b**) Image of a filament in a light microscope. (**c**–**e**,**g**,**h**) SEM images of a filament, where the outlined squares are close-ups. The scale bars are 20 μm in **b**–**d**, 2 μm in **e** and 500 nm in **g**,**h**. (**f**) Diffractograms from a horizontal scan of a filament shown in **b**, where the filament has a diameter of ~30 μm, the beam size is 1.5 × 1.2 μm^2^ (Horiz. × Vert.) and the distance between two diffractograms is 6 μm (the region covered by each diffractogram is indicated with blue rectangles in **b**). The scale bar in **f** is 10 nm^−1^.

After fixation through solvent exchange in acetone and drying with fixed end points—that is, the filament is neither stretched nor allowed to decrease in length during drying—a dry, homogeneous filament is obtained. Figure 5 shows a photo in polarized light, SEM images and micro-focused wide angle X-ray scattering (WAXS) diffractograms of a filament from case B (the other cases are similar except for the degree of alignment). The SEM images Fig. 5c–e,g,h show that the filament is voidfree and has a fairly constant cross-section, although some irregularities are visible. On the fracture surface (Fig. 5e,g), individual fibrils seem to have been pulled out from the structure. One such fibril is indicated with a white arrow in Fig. 5g. From a close-up of the filament surface seen in Fig. 5h, it is observed that the network is so compact that only fibrils on the surface can be identified against a grey background.

Furthermore, the X-ray diffractograms in Fig. 5f, taken at different nonoverlapping positions across the full width of the filament, reveal that the fibril orientation is distinct and does not vary across the filament. Thus, there is no skin-core effect^35^ and the data demonstrate that the filaments have a very homogeneous structure.

**Figure 6:**
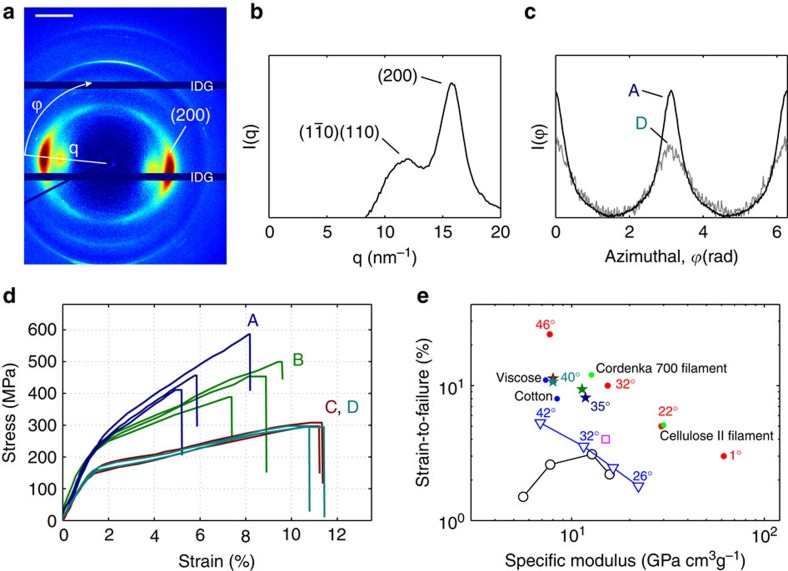
Structure and mechanical properties of the filament. (**a**) Diffractogram of the strongest and stiffest filament (case A), where IDG denotes the intermodular detector gap and the scale bar is 10 nm^−1^. (**b**) Radial integration of the diffractogram shown in **a**. (**c**) Azimuthal integration of the (200) scattering plane of the diffractogram shown in **a** (case A) as well as an integration from case D. (**d**) Stress–strain plot of 10 filaments from cases A–D. (**e**) Strain-to-failure versus specific Young’s modulus for cellulose pulp fibres, filaments and films made from CNF and the hydrodynamically assembled filaments of this study. The angle represents the angle of the CNF towards the filament or fibre axis in case of filaments and towards the tensile testing axis in the case of films.

As mentioned, the filaments are characterized in terms of fibril alignment, stiffness, strength and strain-to-failure. The results from cases A–D are summarized in Table 2. A high-resolution WAXS image together with an identification of the crystal structure, distributions of fibril orientation for cases A and D, stress–strain curves and strain-to-failure versus specific modulus are shown in Fig. 6.

The order parameter is calculated from the azimuthal variation of the scattering peak at *q*=15.8±0.1 nm^−1^, corresponding to the (200) reflection of cellulose I (see Fig. 6b). The (200) reflection is used to quantify the orientation of cellulose crystals and since the crystals are aligned in the direction of the fibril, it can also be used to quantify the fibril orientation^7^. The intensity of this peak as a function of azimuthal angle for cases A and D are shown in Fig. 6c. This is in fact the orientation distribution of the fibrils and through integration of this function, the order parameter is obtained. The mean fibril angle is taken as the constant fibril angle that would result in the same order.

Stress–strain curves for two or three filaments from each case are shown in Fig. 6d. The general shape of the curves are typical for CNF materials^14^,^15^,^16^. Case A (ejected into pure water) is strongest and stiffest; case B (ejected into NaCl) shows a similar initial stiffness but is less stiff as the strain is increased and reaches a lower tensile strength.

## Discussion

As already mentioned, our filaments are stronger and stiffer than previously reported materials made from CNF as can be seen in Figs 1b and 6e, which show the specific ultimate strength, strain-to-failure and Young’s modulus. This cannot be an effect of variations of the CNF raw material since one of the previously reported results (marked with a filled triangle in Fig. 1b) was produced using identically prepared CNF.

From Table 2 it is clear that the filaments from cases C and D have lower structural order compared with cases A and B. Thus, two different ways of controlling the alignment are demonstrated. In case C, the sheath flow concentration of ions is decreased, resulting in gelation further downstream where the order is lower as shown in Fig. 4c, whereas in case D, the acceleration in the channel is decreased and consequently the resulting alignment is decreased as well. The mechanical properties of the filaments from cases C and D are identical, regardless of the method by which the alignment was controlled. The less aligned filaments have a lower stiffness: lower tensile strength but larger strain-at-break compared with the filaments with more aligned fibrils.

Since case A was ejected into deionized water but cases B, C and D were ejected in a NaCl solution, it is probable that there is more NaCl present in the filaments from cases B, C and D. Since case B is as stiff, slightly weaker and has a larger strain-to-failure compared with case A, it can be hypothesized that the presence of salt weakens fibril–fibril interaction.

In fact, our filaments are as strong and stiff as strong cellulose pulp fibres at almost the same mean fibril orientation. An important difference compared with previously reported CNF-based filaments^14^,^15^ is that we form a gel thread in a well-defined low shear environment before further processing. Compared with the previous CNF-based filaments, the filaments prepared by flow focusing can be made considerably thinner.

Comparisons with filaments prepared from regenerated cellulose are also in place. Figure 1b shows that our strongest filament is as strong as the, to our knowledge, strongest commercially available cellulose filament, in spite of the fact that the alignment of our filament is considerable lower (the Cordenka 700 filament consists of extremely aligned cellulose II crystals). The strength of the Cordenka 700 filament is obtained in a highly optimized process including advanced post treatment after spinning. An additional difference is that our filaments consist of the naturally occurring (and ~50% stiffer^36^) cellulose I structure, whereas the filaments from regenerated cellulose result in the cellulose II structure.

Regarding improvements of the mechanical properties, the possibilities of additional treatment (for example, drawing) during drying and of the dried filament remain to be investigated. There might be considerable potential for further improvement, as indicated by the impressive properties of the strongest cellulose filament ever reported^24^. Note that the mechanical properties of the highly optimized filaments prepared from regenerated cellulose coalesce with the properties reported for cellulose fibres from pulp (the filled red circular markers). At this point, it is also necessary to comment on the ecological footprint of different raw materials for cellulose filaments. In order to dissolve and regenerate cellulose, it is necessary to use organic solvents. With CNF as the building blocks, the use of such solvents is avoided.

Thus, the full potential of CNF-based filament have not been realized and optimized post treatment is necessary in order to do so. The homogenous gel thread could be a good starting point for such efforts. The only post treatment used in the present work (drying from 0.3% solid content with fixed end points) can be seen to increase alignment since Fig. 4c shows that the maximum order in the channel for cases A–C is 0.39 (and even lower at the, not exactly determined, point of gelation) and the most aligned dry filaments have an order of 0.5 (cases A and B in Table 2).

Summarizing the conclusions, we have identified a scalable process that produces homogeneous, smooth filaments with aligned nanofibrils from a low concentration dispersion in water. The alignment is achieved by deforming a flow stream of fibrils and the ability of the process is demonstrated by production of cellulose filaments from a dispersion of CNFs. These cellulose I filaments have a specific ultimate strength and specific Young’s modulus in line with natural cellulose pulp fibres at the same fibril alignment, which is a considerable improvement compared with previously reported CNF materials.

The properties of the filaments can be controlled by separating fibril alignment from gelation. A proper understanding of the underlying processes (flow-induced alignment and gelation due to decreased electrostatic repulsion between the fibrils) is necessary in order to achieve this separation. In fact, the process relies on subtle relationships between four timescales. In previously reported work on production of filaments with flow focusing, the necessary separation of timescales has not been at hand.

The properties of cellulose pulp fibres and filaments from dissolved cellulose indicate that high performance filaments, comparable to glass fibres in terms of specific strength and stiffness, can be achieved if the alignment is increased further. The increased alignment must come from process optimization and/or post treatment. The Achilles’ heel is the subtle balance between the governing timescales and the hydrodynamics of flow focusing. Thus, it is clear that detailed and accurate modelling of the process is necessary if this is to be achieved. The reward for success is however substantial. In fact, an environmentally friendly process producing the strongest man-made cellulose material ever may be within reach.

Finally, a few comments on how our findings relate to spider silk and filaments made by carbon nanotubes and/or graphene will be made. It has previously been demonstrated that filaments inspired by spider silk can be produced by flow focusing together with coagulation induced by a change of pH^22^. Until now, the reported strength properties of the artificially made filaments have not reached the impressive values of natural spider silk. In the context of filaments made by carbon nanotubes and/or graphene (possibly mixed with polymers), the nanostructure together with fibril alignment is known to determine the properties (mechanical, electrical and thermal) of the filament^29^,^37^. Considering these results in the light of our present findings, it might be possible to improve and control the properties of both carbon nanotube and graphene filaments as well as artifical silk by designing experiments where the identified timescales are taken into consideration.

## Methods

### CNF and its preparation

CNFs were prepared by liberating fibrils from bleached softwood fibres (Domsjö dissolving, Domsjö AB, Sweden). Before liberation the fibrils were carboxymethylated^38^ to a degree of substitution of 0.1. The fibrils were then liberated from the fibre wall following a protocol described elsewhere^26^. Post-liberation unfibrillated fibre fragments were removed by centrifuging the dispersions at 4750 r.c.f. The protocol generated a transparent dispersion with an approximate CNF concentration of 1 g l^−1^. The dispersion was then allowed to evaporate under mechanical stirring at room conditions to a concentration of 3 g l^−1^.

### Flow set-up

The flow set-up consists of two syringe pumps (WPI, Al-4000), a flow-focusing channel and a bath. The two pumps transfer CNF and NaCl solutions to the channel, where the CNF dispersion is focused, see Fig. 3e. The channel is milled into a poly(methyl methacrylate) (PMMA) plate and sealed with a second PMMA plate on top. In order to prevent leakage, two aluminium plates are placed on either side and screwed together, see Fig. 3a,b. The channel has a square cross-section with the side *h*=1 mm and the three inlets have the length 45 *h*, while the outlet is 50 *h*. The outlet is submerged in a bath.

### Post treatment

A gel filament is produced from the channel and ejected into the bath. The gel filament is then transferred to a water bath in order for the electrolyte to diffuse out of the gel. After 24 h the gel filament is transferred to an acetone bath where it is fixed, after which the gel filament is taken out of the bath and fasten in both ends to dry. After drying, the filaments are assumed to be homogeneous cellulose materials and have a density^4^ of 1.5 g cm^−3^.

### *In situ* SAXS measurements

SAXS measurements were performed in a slightly modified flow set-up, the channel was cut out of a 1 mm thick stainless steel plate that was sandwiched between two Kapton windows. CNF was used as core fluid while deionized water was used as sheath fluid. Owing to the long exposure times and limited access to the experimental hutch, clogging prevented SAXS measurements with gelation. The diffraction measurements were performed at the P03 beamline^39^,^40^ at the PETRA III storage ring at DESY in Hamburg, Germany. SAXS measurements were performed in a transmission geometry with an X-ray wavelength 
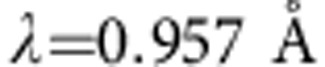
 and sample-to-detector distance of 8,422 mm. The beam size was 24 × 11 μm^2^ (Horiz. × Vert.) and a single-photon counting detector (Pilatus 1 M, Dectris) having the pixel size of 172 × 172 μm^2^ was used to record the scattering patterns.

The quantification of the SAXS patterns was performed by first transforming the diffractogram into a rectangular image with the scattering vector, *q* (defined as 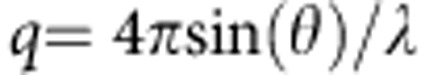, where *θ* is the scattering angle), and the azimuthal angle, *φ*, as coordinates. At each *q*-value the distribution was normalized with the highest intensity. A background intensity was removed under the assumption that the highest oriented case did not have any particles aligned in the direction perpendicular to the flow (the value at *φ*=90 was set to zero) for each *q*-value. The final orientation (intensity) distributions were then found by taking the mean between 0.6<*q*<0.9 nm^−1^, for each measurement.

The alignment of the CNF was quantified by converting the orientation distributions to the order parameter^41^, *S*, defined as:


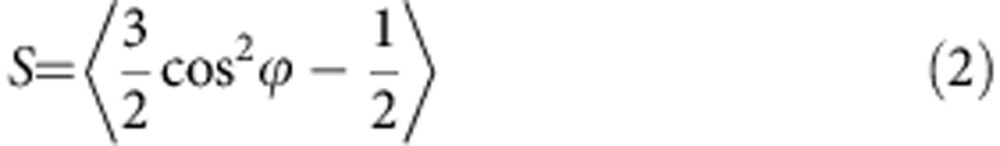


where 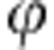 is the azimuthal angle in a diffractogram. Expanding the average gives:


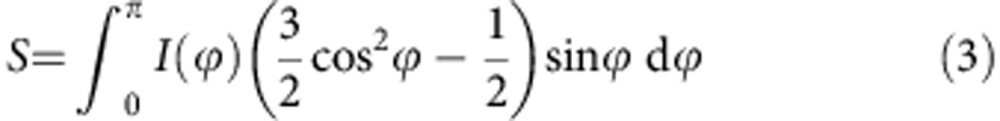


which is normalized according to:


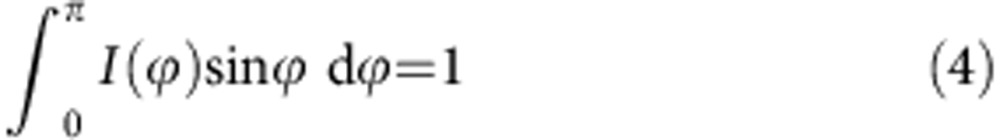


where 
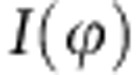
 is the intensity distribution along a constant *q*-value.

### Filament characterization

The polarized microscopy images in Figs 4a and 5a and were acquired using a microscope (Nikon SMZ 1500) with a camera (Basler, piA1900-32gm), where a light source (Schott, KL2500 LCD) was placed behind the channel and filament in Figs 4a and 5a and, respectively. The polarization filters were oriented with a 45° angle with respect to the flow in Fig. 4a and to the horizontal axis in Fig. 5a.

The tensile tests were carried out with a Deben Micro tester and a 50 N load cell. The filaments were left in 50% relative humidity and 298 K for 24 h, before the tensile tests, which were performed in the same environment. The span length was 7–10 mm and the measurements were performed at 0.2 mm min^−1^. The cross-section of each filament was assumed to be circular and was determined with a light microscope. The number of samples was three for cases A and B and two for cases C and D.

For the SEM, a 10-nm gold–palladium layer was sputtered (Cressington Scientific Instruments Ltd, UK) on the surface of the filament. The imaging of the filaments was performed by using a Hitachi S-4800 Field Emission-Scanning Electron Microscope (Hitachi, Japan) operated in the secondary electron imaging mode at an acceleration voltage of 1 kV.

The WAXS measurements were performed at the P03 beamline^39^,^40^ at the PETRA III storage ring at DESY. WAXS measurements were performed in transmission geometry using three different optical configurations. For the measurements performed across the filament (Fig. 5) an X-ray wavelength of 0.809 Å and a sample-to-detector distance of 238 mm was used and the scattering patterns were recorded using a CCD detector (Photonic Science) with a pixel size of 91.8 × 91.8 μm^2^. The beam size in this case was 1.5 × 1.2 μm^2^ (Horiz. × Vert.). The second configuration was to determine the alignment inside the filaments from cases C and D; here, the X-ray wavelength was 0.827 Å and a sample-to-detector distance of 181.4 mm, with the same detector and beam size as above. The measurements for the quantification of the alignment of cases A and B (Figs 1 and 6) were performed at an X-ray wavelength of 0.954 Å. The sample-to-detector distance was set to 104 mm and the beam size was 24 × 17 μm^2^. In this case, a Pilatus 300-k detector (Dectris) whose pixel size is 172 × 172 μm^2^ was used.

The alignment was quantified with the order parameter, *S*, in the same manner as with the SAXS patterns. Here, however, the distribution 
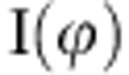
 is taken at the (200) reflection in the diffractogram. The resulting *S*-values were converted back into an angle assuming the distribution to be a *δ*-function in equation (3). The *S*-values given in the study by Sehaqui *et al*.^16^ were converted into degrees in the same manner in order to compare with the cellulose pulp fibres.

### Rotational diffusion measurement

The rotational diffusivity, *D*_rot_, was estimated by a combination of flow orientation and the polarized light set-up. The fibrils of a CNF dispersion were aligned (by water) in the flow focusing cell. By stopping the flow (turning the pumps off and plugging the exit) and measure the decay of the light intensity, *D*_rot_ could be estimated. Assuming a biaxial symmetry, the rotational diffusivity *D*_rot_ is related to the light intensity, *I*, the birefringence, Δ*n*, the depth of the material, *d* as follows:


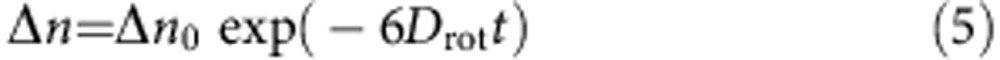


where *t* is the decay time from when the flow has been stopped^42^,^43^.

The measured intensity is then proportional to *D*_rot_ as:


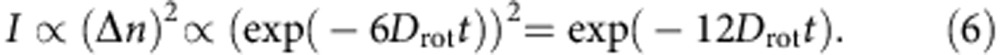


In our case, *D*_rot_=0.04 rad^2 ^s^−1^ was obtained.

## Author contributions

K.M.O.H., A.B.F., F.L., M.K., L.W. and L.D.S. came up with the original idea of producing CNF filaments by combining alignment and the disp-gel transition in a flow-focusing set-up. K.M.O.H., F.L. and L.D.S. designed the flow and K.M.O.H. set it up. K.M.O.H. and A.B.F. prepared the CNF. K.M.O.H. produced the filaments and discussed with A.B.F., F.L., M.K., L.P.W., L.D.S. and L.W. during set-up and execution of the experiments. K.M.O.H. and A.B.F. performed the SEM. K.M.O.H. performed the tensile testing. S.Y., C.K., S.V.R. and G.S. designed and performed the nanofocus WAXS measurements. K.M.O.H., S.Y., A.B.F., F.L., M.K., L.P.W. and L.D.S. performed the micro-focus WAXS and SAXS measurements and C.K., G.S. and S.V.R. assisted them. K.M.O.H. prepared Figs 1 and 3–6. ABF prepared Fig. 2. K.M.O.H., F.L. and L.D.S. wrote most of the paper. A.B.F. wrote the part about preparation of CNF. S.Y. wrote the part about the diffraction measurements.

## Additional information

**How to cite this article:** Håkansson, K. M. O. *et al*. Hydrodynamic alignment and assembly of nanofibrils resulting in strong cellulose filaments. *Nat. Commun.* 5:4018 doi: 10.1038/ncomms5018 (2014).

## Figures and Tables

**Table 1:** Filament production conditions reported in present work.

**Case**	**Core flow rate,** ***Q***_**1**_ **(mm**^**3 **^**s**^**−1**^)	**Sheath flow rate,** ***Q***_**2**_ **(mm**^**3 **^**s**^**−1**^)	**Acceleration,** ***Q***_**2**_**/*****Q***_**1**_	**NaCl conc in sheath flow (mM)**	**NaCl conc in bath (mM)**
A	6.5	7.5	1.15	100	*0*
B	6.5	7.5	1.15	100	100
C	6.5	7.5	1.15	*50*	100
D	6.5	*4.5*	*0.69*	100	100

Conc, concentration.

The outlier parameter(s) in cases A, C and D is/are italic.

**Table 2:** Properties of the dried filaments.

**Case**	**Radius,** ***r*** **(μm)**	**Tensile strength,** ***σ*_c_** **(MPa)**	**Modulus,** ***E*** **(GPa)**	**Strain-to-failure,** **ε_c_** **(%)**	**Order parameter,** ***S***	**Mean fibril angle (°)**
A	14 (1.5)	490 (86)	17.6 (0.7)	6.4 (1.6)	0.50 (0.01)	35 (0.4)
B	11 (0.2)	445 (60)	18.0 (0.8)	8.6 (1.1)	0.50 (0.01)	35 (0.5)
C	16 (1)	300 (20)	12.4 (0.5)	11.2 (0.1)	0.38 (0.02)	40 (1)
D	19 (1)	295 (19)	12.8 (0.5)	11.1 (0.4)	0.39 (0.02)	40 (1)

The mean and s.d. (in parenthesis) are given.

The number of measured samples is three for cases A and B and two for cases C and D.
